# Synthesis and Characterization of a Novel Zinc-Based Metal-Organic Framework Containing Benzoic Acid: A Low-Toxicity Carrier for Drug Delivery

**DOI:** 10.5812/ijpr-136238

**Published:** 2023-07-14

**Authors:** Xiaomei Ye, Mengmeng Xiong, Kangrui Yuan, Wei Liu, Xiaoqun Cai, Yu Yuan, Yanghang Yuan, Youfa Qin, Dudu Wu

**Affiliations:** 1 Department of Clinical Pharmacy, SSL Central Hospital of Dongguan City, Affiliated Dongguan Shilong People’s Hospital of Southern Medical University, Dongguan, China; 2 NO.2 High School of East China Normal University, Shanghai, China; 3 College of Chemical Engineering Nanjing Forestry University, Nanjing, China; 4 The First Dongguan Affiliated Hospital of Guangdong Medical University, Dongguan, China; 5 School of Pharmacy, Guangdong Medical University, Dongguan, China

**Keywords:** Keywords: Metal-Organic Frameworks, Drug Delivery Systems, Benzoic Acid, Doxorubicin

## Abstract

In recent years, metal-organic frameworks (MOFs) have gained attention in the biomedical field, particularly as drug carriers for treating tumors. Therefore, we decided to synthesize a novel benzoic acid Zn-based MOF and study the Zn-based MOFs' drug-delivery properties and the drug-delivery system's anticancer effects. This study successfully synthesized a zinc-based MOF using solvent thermal synthesis. The crystal structure of a Zn-based MOF was investigated using thermogravimetric analysis, X-ray diffraction, and Fourier transform infrared spectroscopy. Subsequently, the results of UV spectrophotometry showed that Doxorubicin was successfully loaded with a loading amount of 33.74%. Furthermore, the drug release experiments demonstrated that the Zn-based MOF was pH-sensitive, releasing more at a pH of 3.8 than at pH 5.8 or 7.4. Finally, the Zn-based MOF loaded with drugs exhibited high antitumor activity against HepG2 cells while demonstrating remarkably low toxicity to normal cells (LO2). Taken together, these results demonstrate that the Zn-based MOF has the potential to serve as a carrier in the field of drug delivery systems.

## 1. Background

Doxorubicin (DOX) is a chemotherapy drug commonly used to treat various types of cancer, including breast and ovarian cancer. It has been widely used due to its broad-spectrum antitumor activity (1). However, its potent cytotoxicity and adverse side effects have restricted its clinical usage. Therefore, an effective drug delivery system was needed to reduce the drug's toxicity and side effects while maintaining its therapeutic efficacy.

Metal-organic frameworks (MOFs) are a rapidly emerging class of hybrid materials that combine metal ions and organic ligands. They have been widely used in various applications, including gas adsorption and separation, gas storage, catalysis, and photoelectromagnetic materials. Also, MOFs are highly attractive due to their unique characteristics, such as large pore sizes, remarkable surface areas, and giant pore volumes (2-5). In recent years, MOFs have emerged in the biomedical field (6-12), especially as drug carriers for treating tumors (13-15). For instance, Ni et al. reported two Hf-based MOFs, designed as carriers for combined checkpoint blockade immunotherapy and radiotherapy, showing high antitumor ability against CT26-induced colorectal cancer model in mice (16). In cancer treatment, MOFs have played an important role as drug carriers. They have several advantages over traditional drug carriers, including high specific surface area, controllable pore size, adjustable surface chemistry, and good biocompatibility. These features enable MOFs to enhance drug solubility and stability and release drugs sustainably, prolonging their plasma half-life (17). As a result, MOFs can improve the therapeutic efficacy of drugs and reduce their side effects.

Zinc-based MOFs have a broad range of applications as drug carriers. They are gaining increasing attention in the field of drug delivery due to their exceptional chemical stability and biocompatibility. The high thermal and chemical stability of zinc-containing MOFs is determined by zinc ions' electronic structure and chemical properties, which allows stability to be maintained under different environmental conditions. Additionally, MOFs composed of zinc typically have larger surface areas and more porous structures, which can be used for drug adsorption and release due to the high spatial coordination of zinc ions (18). The pore structure and size of zinc-based MOFs can be controlled by adjusting the structure and length of the ligand, enabling specific drugs to be precisely loaded. Furthermore, good biocompatibility is exhibited by zinc-based MOFs as drug carriers because zinc is a necessary trace element widely present in the human body.

Moreover, MOFs containing aromatic carboxylic acids, such as benzoic acid, as organic ligands exhibit the facile formation of secondary building units (SBUs) and demonstrate exceptional thermal stability (19, 20). For example, Qin et al. synthesized a Ni-based MOF using 3-nitro-4-(pyridine-4-yl) benzoic acid as ligands. Meanwhile, the MOFs started decomposing beyond 364°C, showing remarkably high structural stability due to their 3D supramolecular frameworks (21). Jin et al. investigated an uncommon 3D MOF based on benzoic acid, which possessed excellent stability and diverse function compared with MOFs formed by pyridine (ligand) with N as the coordination atom (22). This might attribute to oxygen atoms being more reactive chemically than nitrogen atoms.

## 2. Objectives

In this study, a Zn-based MOF (Zn_2_(DABA)_3_(CH_3_O), DABA=4-dimethylaminobenzoic acid, DAZ) was synthesized with 4-dimethylaminobenzoic acid and Zn(NO_3_)_2_·6H_2_O by the solvothermal method approach in mixed solvents (N, N-dimethylformamide:methanol=3:2). Then, the loading of DOX into DAZ was also investigated in this research. Finally, the properties of the drug delivery system for DAZ@DOX were tested, and the preliminary anticancer effects of DAZ and DAZ@DOX were revealed through CCK-8 assays. The new DAZ can provide a novel approach for designing MOF carriers that exhibit pH-responsive release and targeted delivery to tumors.

## 3. Methods

### 3.1. Materials and Reagents

All solvents and starting materials were commercially available and used without further purification. In each step during the experiment, the double-steamed deionized water was utilized. Besides, N,N-dimethylformamide (DMF), and Zn(NO_3_)_2_·6H_2_O were obtained from Tianjin Kermel Chemical Reagent Co., Ltd. Also, 4-dimethylaminobenzoic acid (DABA) was purchased from Shanghai Macklin Biochemical Technology Co., Ltd. Doxorubicin hydrochloride (DOX) was bought from Sigma-Aldrich. Cytotoxicity Assay Kit-8 (CCK8) was bought from Solarbio Life Sciences.

### 3.2. Preparation of DAZ

We synthesized DAZ via the solvothermal approach within a 20 mL solvent mixture, including 12 mL of DMF and 8 mL of methanol (3:2). Afterwards, a mixture of 1.0 mmol of Zn(NO_3_)_2_·6H_2_O (297.4 mg) and 1.0 mmol of 4-dimethylaminobenzoic acid (165.2 mg) was loaded into a Teflon-lined autoclave (20 mL). Then, the autoclave was heated from room temperature to 120°C at a rate of 10°C per hour, and the temperature was maintained at 120°C for three days. After 3 days of insulation, the sample was cooled until room temperature at 5°C/h. Finally, the colorless crystal was harvested and centrifuged at 4000 rpm for 30 seconds with 3 mL ethanol.

### 3.3. Characterization of DAZ

An X-ray diffractometer (Bruker D8, Bruker Company, USA) was utilized to obtain the single X-ray diffraction (XRD) patterns. The Hitachi S-4700 cold field-emission scanning electron microscope (Tokyo, Japan) was used to observe the surface morphology of DAZ. The structure of DAZ was drawn by the OLEX2. The specific surface area and pore volume of DAZ and DAZ@DOX were tested using ASAP2020 M+C surface area. The N2 adsorption-desorption analysis was conducted to evaluate the change in the specific surface area of the drug carrier. The thermogravimetric analyzer (PerkinElmer, USA) was employed for thermogravimetric analysis (TGA) under a nitrogen atmosphere (heating rate: 10°C/h). Besides, the samples were dissolved in ethanol. The liquid supernatant was collected for further drug release experiments. UV-visible absorption spectroscopy was obtained by a UV-visible spectrophotometer (Lambda 35, PE, USA). An FT-IR spectrophotometer (Nicolet Magna 550II) was used to obtain the spectra (4000 - 500 cm^-1^). The sample size was measured by a nanotrac wave II nanoparticle-size analyzer.

### 3.4. Drug Loading

We added 0.2 g DAZ into a beaker with Doxorubicin hydrochloride (20 mL, 1.0 g/L), placed at room temperature and dark room. The absorbance of the supernatant was measured via a UV spectrophotometer (absorption wavelength, 460 nm) at different periods of 24 h, 48 h, and 72 h, respectively. The corresponding concentration could be calculated based on the working curve of DOX (A = 14.95562C-0.06671, A: Absorbance; C: Concentration). Besides, the UV-vis absorption spectrophotometry storage capacity of DAZ for UV-vis absorption spectrophotometry was studied by measuring the absorption at a wavelength of 460 nm.

### 3.5. Release of DOX

Experiments were conducted to release DAZ loaded with doxorubicin hydrochloride in buffer solutions with pH values of 3.8, 5.8, and 7.4. The dialysis bag containing DAZ@DOX (0.1 g) and 10 mL of PBS was sealed at both ends. Afterward, the beaker, along with the aforementioned dialysis bag, PBS (200 mL), and magnetic stirrer, was placed on the magnetic stirrer and stirred slowly. The absorbance of the buffer solution in the beaker was measured at regular intervals while replenishing it with the same volume of fresh buffer solution. A drug can be considered fully released when the absorbance value remains essentially unchanged. All experiments were conducted in triplicate to ensure the accuracy and reproducibility of the results.

### 3.6. Cytotoxicity Assay

To investigate the anticancer effect and toxicity of DAZ@DOX, the CCK8 method was used to study Zn(NO_3_)_2_, DABA, DAZ, and DAZ@DOX. Cells (LO2 and HepG2) were inoculated into 96-well microplates and incubated at 37°C in a 5% CO_2_ incubator for 24 hours. Afterward, Zn(NO_3_)_2_, DABA, DAZ, and DAZ@DOX were added to the cell culture medium at concentrations of 0, 1, 5, 10, and 25 mg/mL. After a 24-hour incubation period, the cell culture medium was removed. Afterward, the cells were washed with PBS, and their viability was assessed using the CCK-8 assay. All experiments were conducted in triplicate to ensure the accuracy and reproducibility of the results.

## 4. Results and Discussion

### 4.1. Crystal Structure of DAZ

#### 4.1.1. X-ray Diffraction

**Figure 1.**
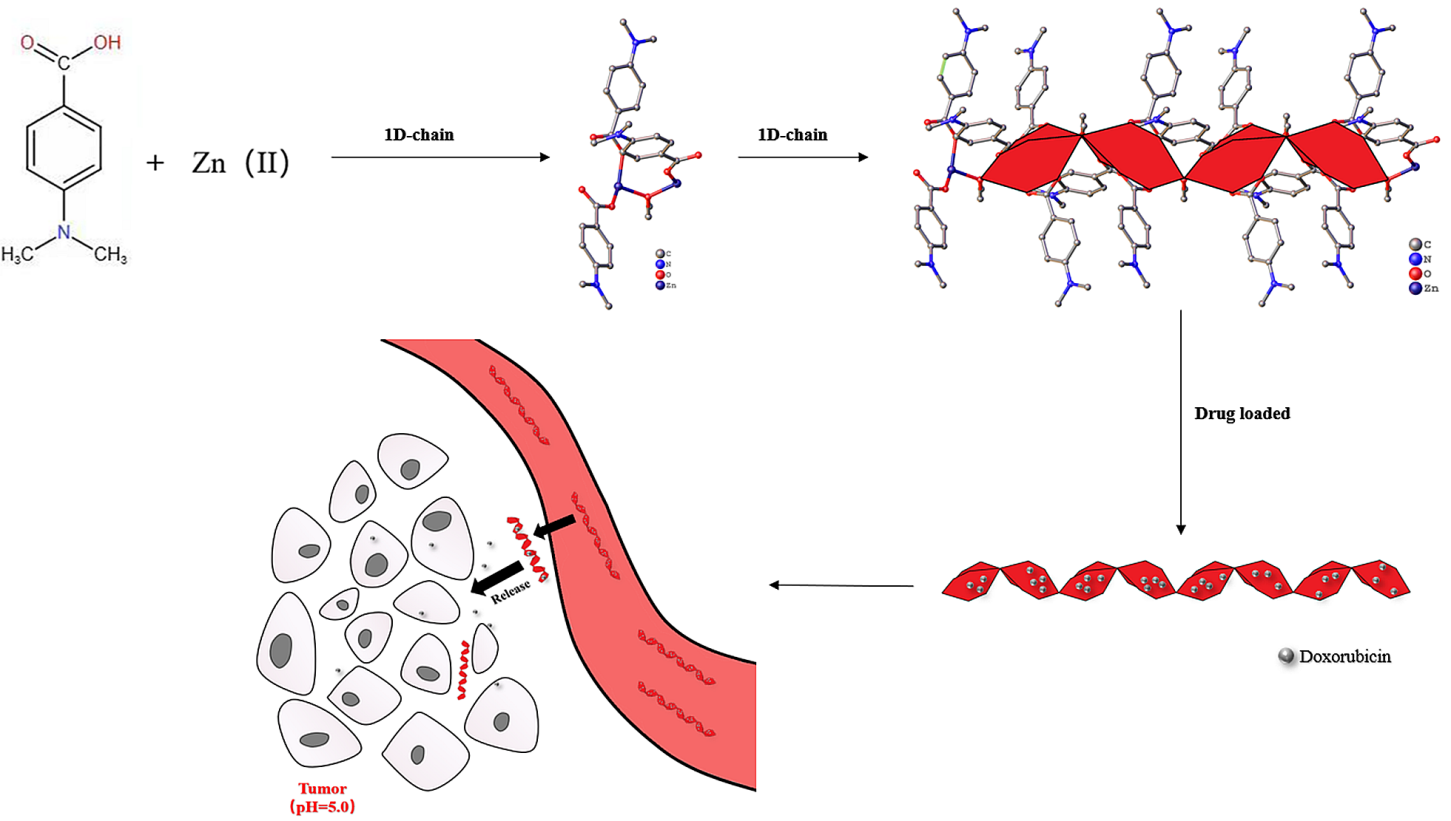
Schematic representation of the synthesis and crystal structure of DAZ (CCDC: 2041878) with a displayed cage-like 1D pore channels and 1D-chains

**Figure 2.**
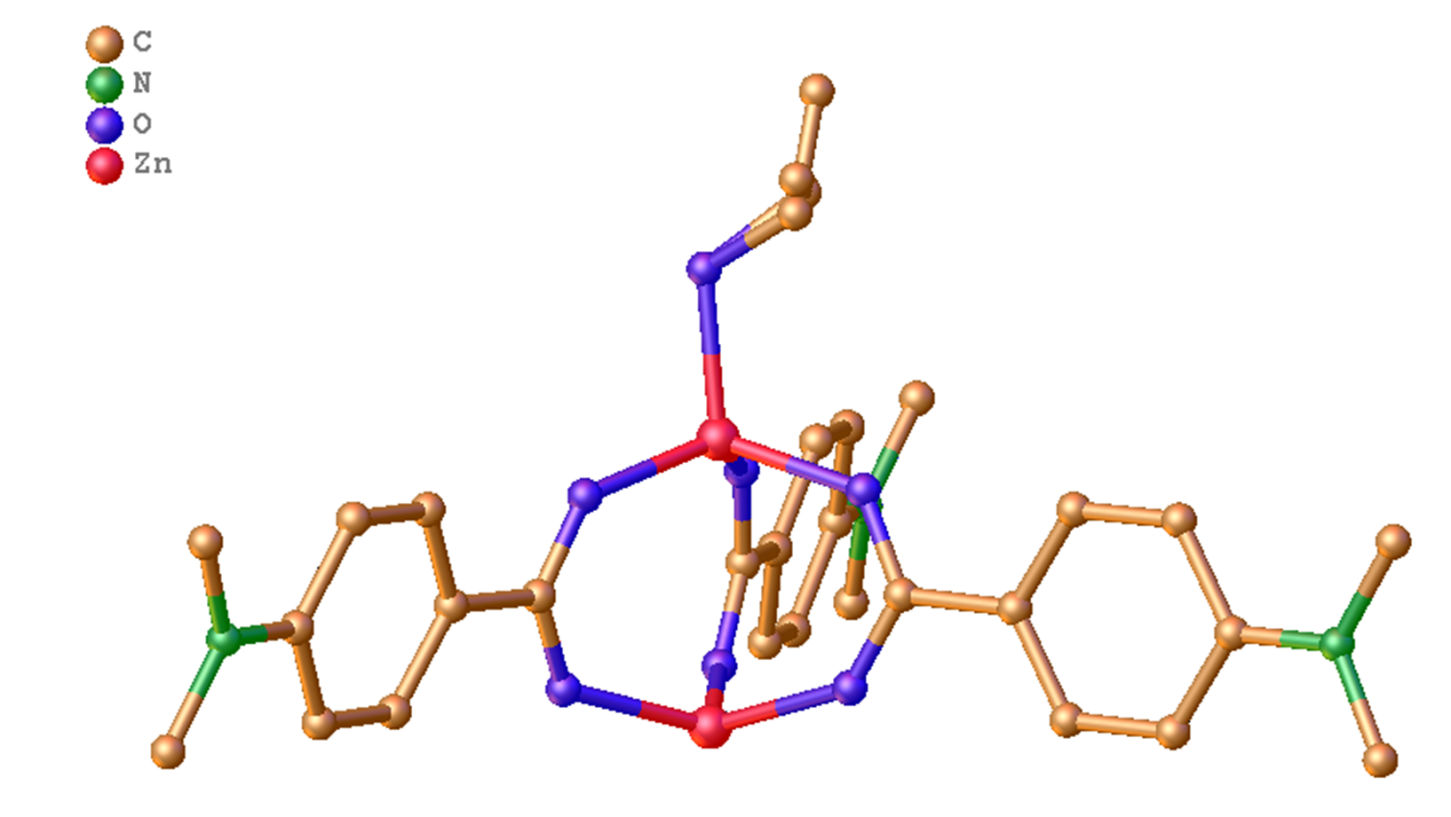
Structure of complex DAZ

**Figure 3.**
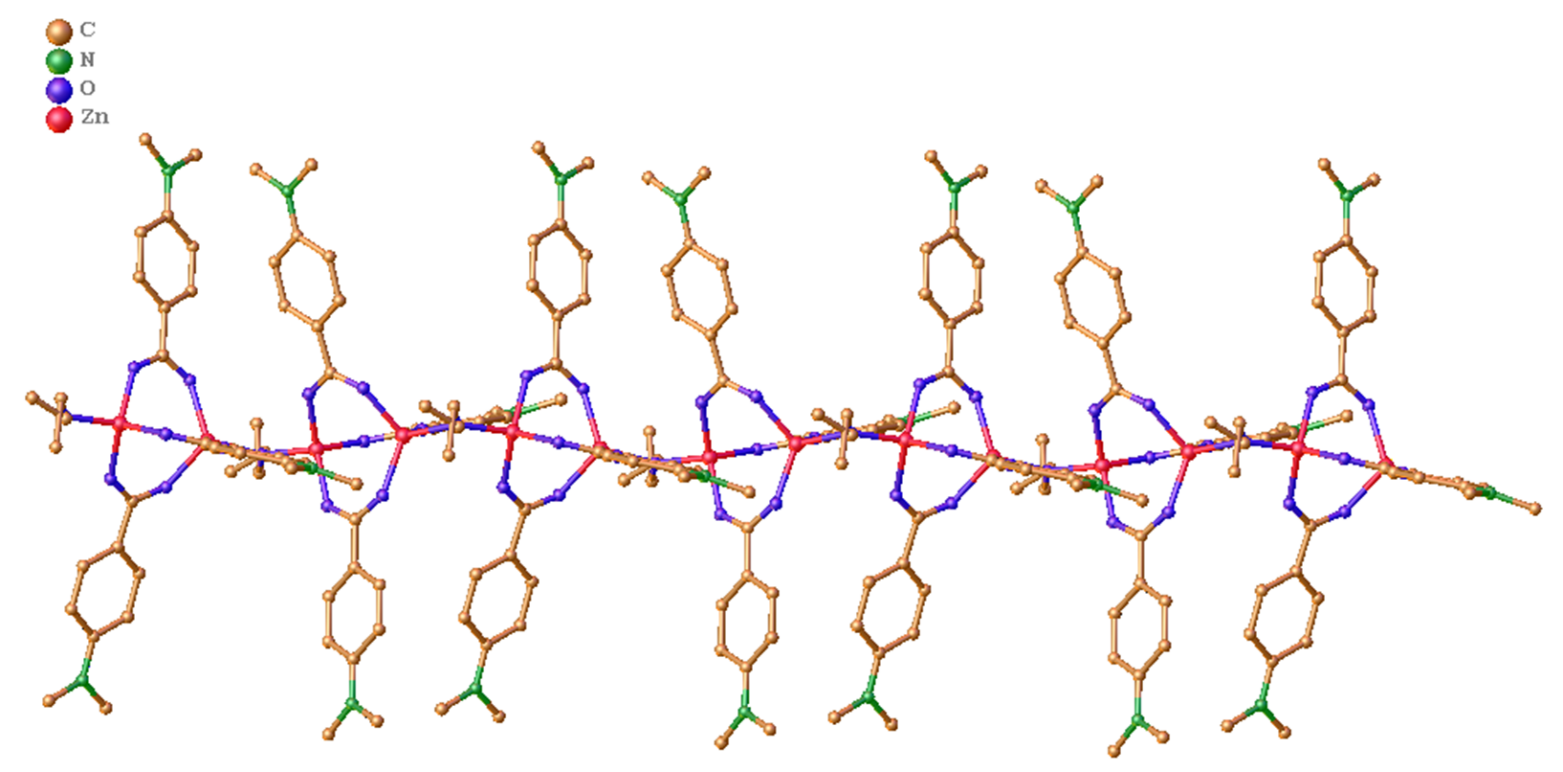
One-dimensional chain structure of DAZ

The crystalline structure of DAZ was investigated using XRD. DAZ was crystallized in the monoclinic system with the space group P 1 21/n 1. Furthermore, Zn^2+^ is coordinated by six atoms, including one oxygen atom from methanol and three carboxyl groups of DABA (Figure 1), forming an octahedral coordination geometry. The fundamental building unit contained two Zn^2+^ cations (located on the symmetry center), three DABA ligands, and one methanol molecule. The structure contains two crystallographically independent Zn(II) centers, each with a different coordination number. The Zn1 center had three connections, while the Zn2 center had two connections. Furthermore, the structure of DAZ was also stabilized by a π-π interaction between the benzene ring in DABA. The independent crystallographic units are connected to each other through the ligand, forming a 1D chain (refer to Figures 2 and 3). The detailed data for DAZ is also presented in Tables 1 and 2.

**Table 1.** Crystallographic Data and Structure Refinement for DAZ ^a^

Empirical Formula	C_28_H_33_N_3_O_7_Zn_2_
**Formula weight**	654.31
**Temperature (K)**	296.15
**Wavelength (Å)**	0.71073
**Crystal system**	Monoclinic
**Space group**	P 1 21/n 1
**a (Å)**	16.3082 (15)
**b (Å)**	11.8462 (11)
**c (Å)**	16.6436 (16)
**α (°)**	90°
**β (°)**	100.628 (2)°
**γ (°)**	90°
**Unit cell volume (Å3)**	3160.2 (5)
**Z**	4
**Density (calculated)**	1.375 mg/m^3^
**Absorption coefficient**	1.563 mm^-1^
**F(000)**	1352
**Crystal size**	0.11 x 0.1 x 0.09 mm^3^
**Theta range for data collection**	2.490 to 27.540°
**Index ranges**	-21 ≤ h ≤ 15, -15 ≤ k ≤ 14, -21 ≤ l ≤ 21
**Reflections collected**	18682
**Independent reflections**	7166 [R(int) = 0.0271]
**Completeness to theta = 25.242°**	99.7%
**Absorption correction**	Semi-empirical from equivalents
**Max. and min. transmission**	0.7456 and 0.5571
**Refinement method**	Full-matrix least-squares on F^2^
**Data/restraints/parameters**	7166 / 20 / 378
**Goodness-of-fit on F2**	1.031
**Final R indices [I > 2 sigma (I)]**	R_1_ = 0.0403, wR_2_ = 0.1121
**R indices (all data)**	R_1_ = 0.0619, wR_2_ = 0.1257
**Extinction coefficient**	n/a
**Largest diff. peak and hole**	1.216 and -0.591 e.Å-3

^a^ R = ∑(F_o_ -F_c_)/∑(F_o_); wR_2_ = (∑[w(Fo^2^-Fc^2^)^2^]/∑(Fo^2^)^2^)^1/2^.

**Table 2.** Bond Lengths (Å) and Angles (°) ^a^

Bond	Lengths (Å) and Angles (°)
**Zn(1)-O(1) →**	1.9272 (19)
**Zn(1)-O(4) →**	1.943 (2)
**Zn(2)-O(1) →**	1.9276 (19)
**Zn(2)-O(2) →**	1.938 (2)
**O(2)-C(1) →**	1.261 (4)
**O(6)-C(20) →**	1.260 (4)
**N(1)-C(5) →**	1.378 (4)
**N(1)-C(7) →**	1.447 (5)
**C(9)-C(10) →**	1.382 (5)
**C(10)-C(11) →**	1.389 (6)
**N(2)-C(12) →**	1.433 (7)
**O(1)-C(13) →**	1.507 (12)

^a^ Symmetry transformations used to generate equivalent atoms: #1 -x+1, -y+1, -z #2 x+1, y, z #3 x-1, y, z.

**Figure 4.**
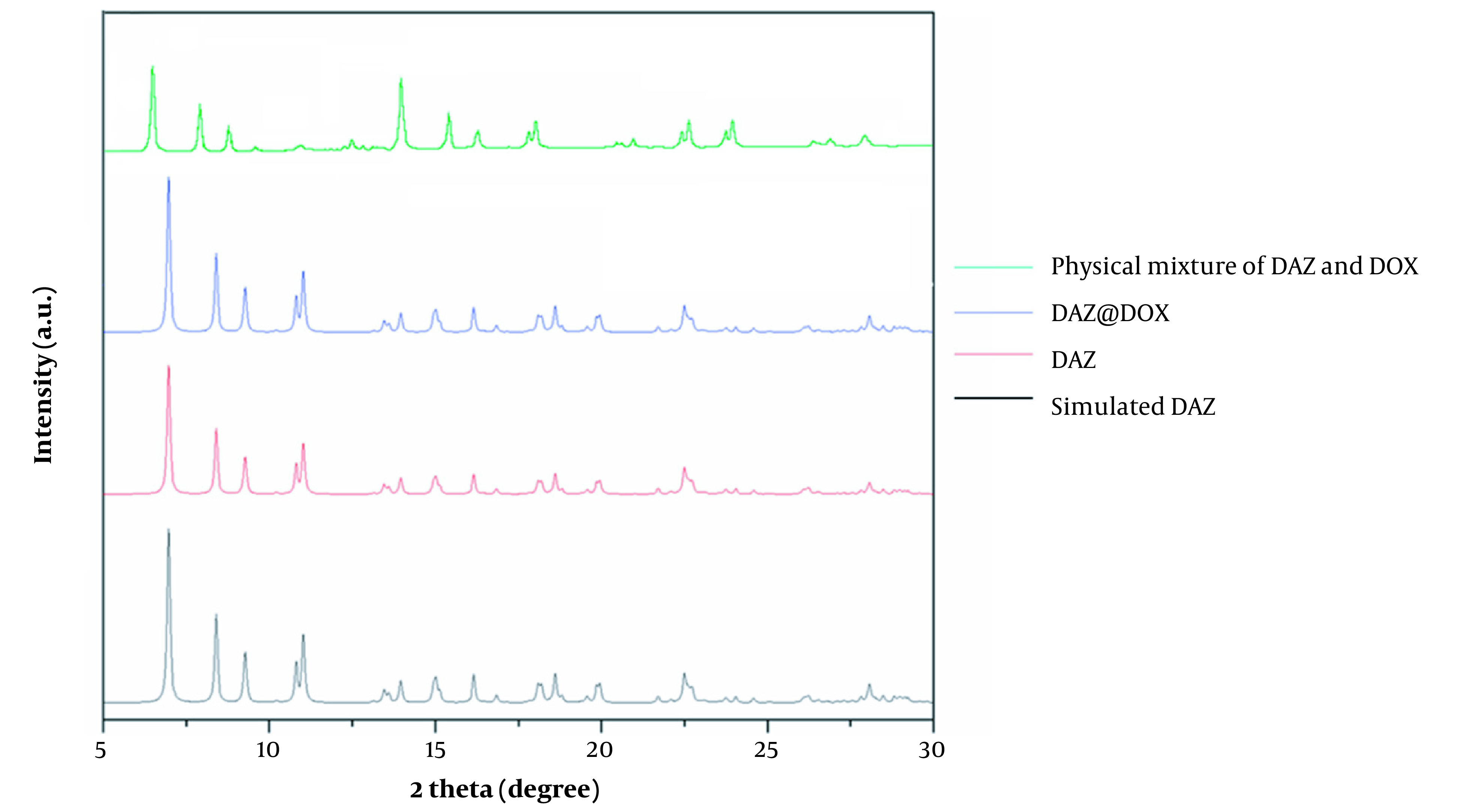
X-ray diffraction (XRD) patterns of the physical mixture of DAZ and DOX, DAZ@DOX, DAZ, and simulated DAZ

Figure 4 depicts the characteristic peaks observed in XRD corresponding to a physical mixture of DAZ and DOX, DAZ@DOX, DAZ, and simulated DAZ. The XRD curves of DAZ and stimulated DAZ were similar, which indicates the successful synthesis of DAZ with remarkably high purity. The XRD characteristic peaks of DAZ before and after drug loading were almost identical, indicating that the structure of DAZ remained unchanged.

#### 4.1.2. Surface Analysis

**Figure 5.**
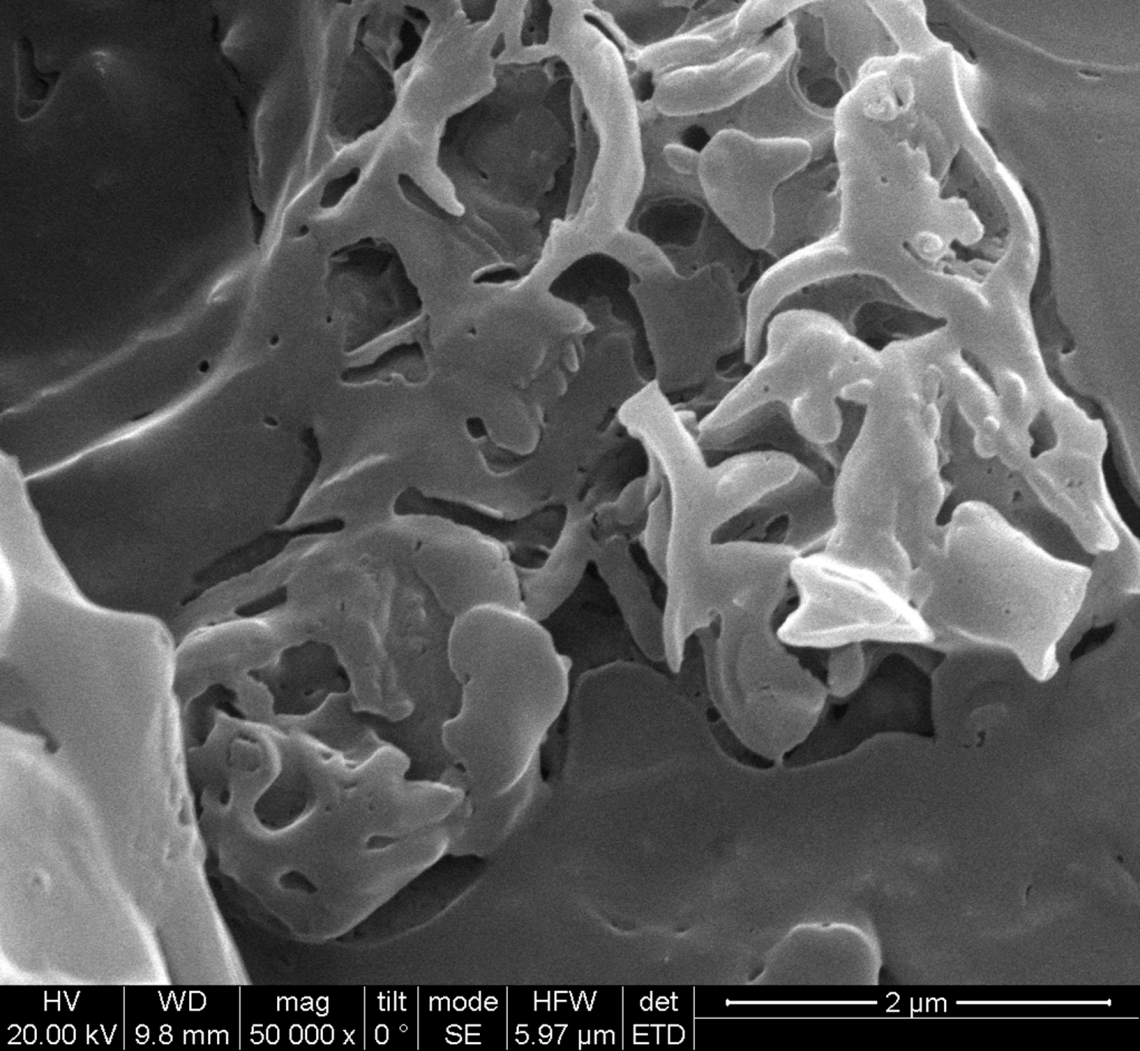
The SEM image of DAZ

**Figure 6.**
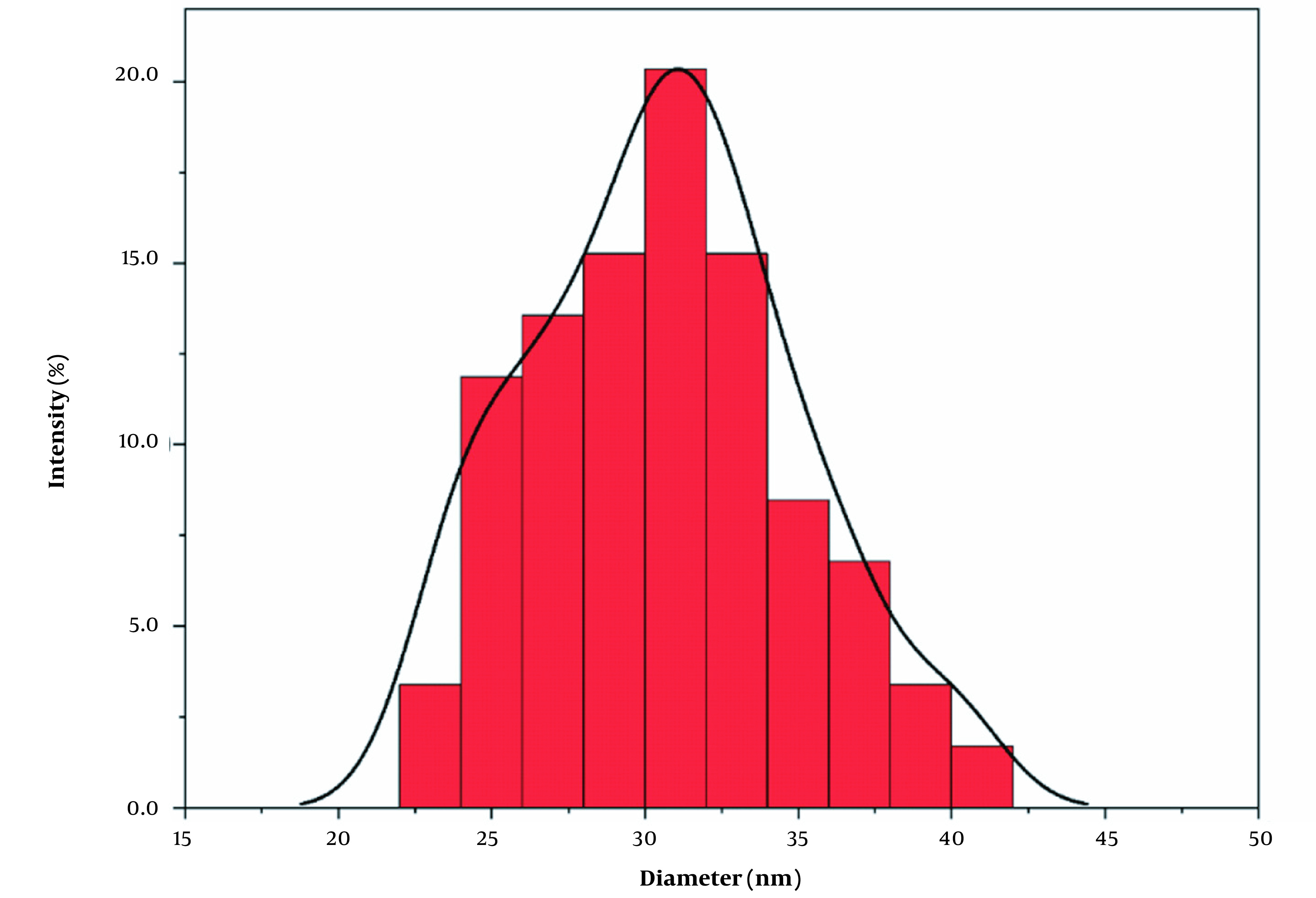
Nanoparticle size measurement result of DAZ

The SEM image of DAZ with a vesicular structure is depicted in Figure 5. According to the nanoparticle size analyzer, the size of DAZ nanoparticles was measured to be approximately 31 nm (n = 50), as shown in Figure 6. Using Scherrer's equation (D = k (λ/β cos θ)), the mean size of DAZ can be calculated to be around 30 nm. In this case, "k" represents the constant (0.89), "λ" represents the wavelength of the X-ray (0.154 nm), "θ" represents the half diffraction angle, and "β" represents the half maximum full width. The results obtained from the nanoparticle size analyzer confirmed the crystallite size calculated using the Scherrer equation.

The results of N2 adsorption-desorption tests indicate that DAZ has a specific surface area (S_BET_) of 1532.58 m^2^/g, a pore volume of 0.36 cm^3^/g, and a pore size of 4.107 nm (refer to Table 3). These results reveal that DAZ has great potential as a drug carrier. After drug loading, the S_BET_, pore volume, and pore size were 1380.01, 0.28, and 3.471, respectively. The surface area and pore volume of DAZ@DOX decreased compared to DAZ, indicating the successful loading of DOX and its occupation of a certain volume within the material.

**Table 3.** Texture Parameters and Drug-Loaded Capacity of DAZ Mesoporous Molecular Sieves (a) Before and (b) After Loading Doxorubicin

Sample	S_BET_ (m^2^/g)	Pore Volume (cm^3^/g)	Pore Size (nm)	DOX Loading Amount (%)
**a**	1532.58	0.36	4.107	-
**b**	1380.01	0.28	3.471	33.74

Abbreviation: DOX, doxorubicin.

#### 4.1.3. TGA

**Figure 7.**
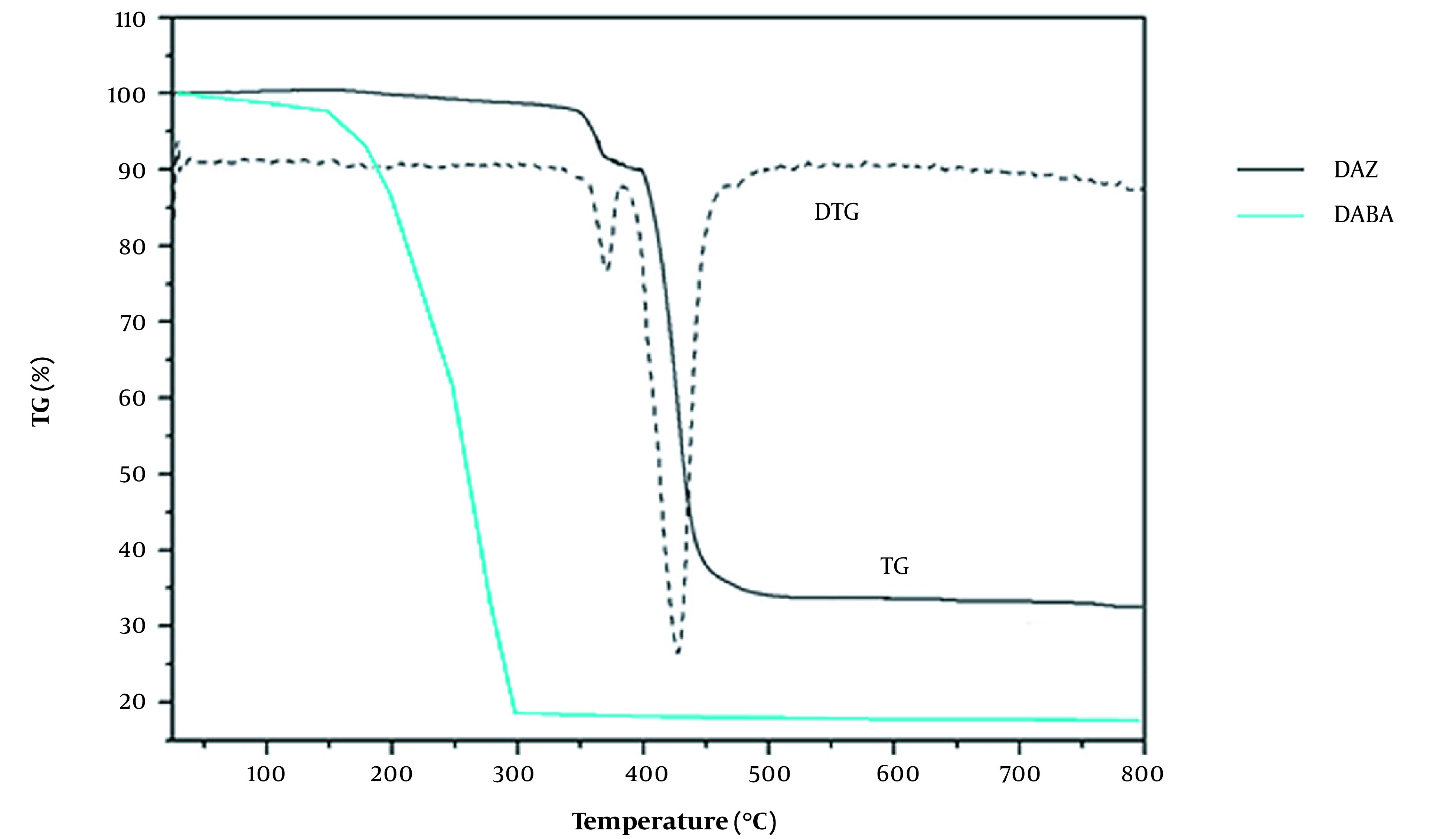
Thermogravimetric curve of DAZ and DABA

The thermal stability of DAZ and DABA was studied using TGA (Figure 7). The TGA curve of DAZ showed the first stage of weight loss occurring in the temperature range of 150 to 300°C, with a weight loss of 4.9% (calculated as 4.87%). This suggests that a coordinated methanol molecule may have been lost. The weight loss during the second stage, which occurred between 330°C and 400°C, was attributed to removing organic ligands (calculated at 70.37%; found at 70.45%). The observed third weight loss of 24.65% corresponds to the generation of ZnO (calculated at 24.76%). The weight loss (around 150 ~ 300°C) was observed for the DABA TGA curve, indicating distinct thermal instability characteristics. Significant differences in the TGA curves of DAZ and DABA demonstrate the successful synthesis of the MOF.

#### 4.1.4. IR Analysis

**Figure 8.**
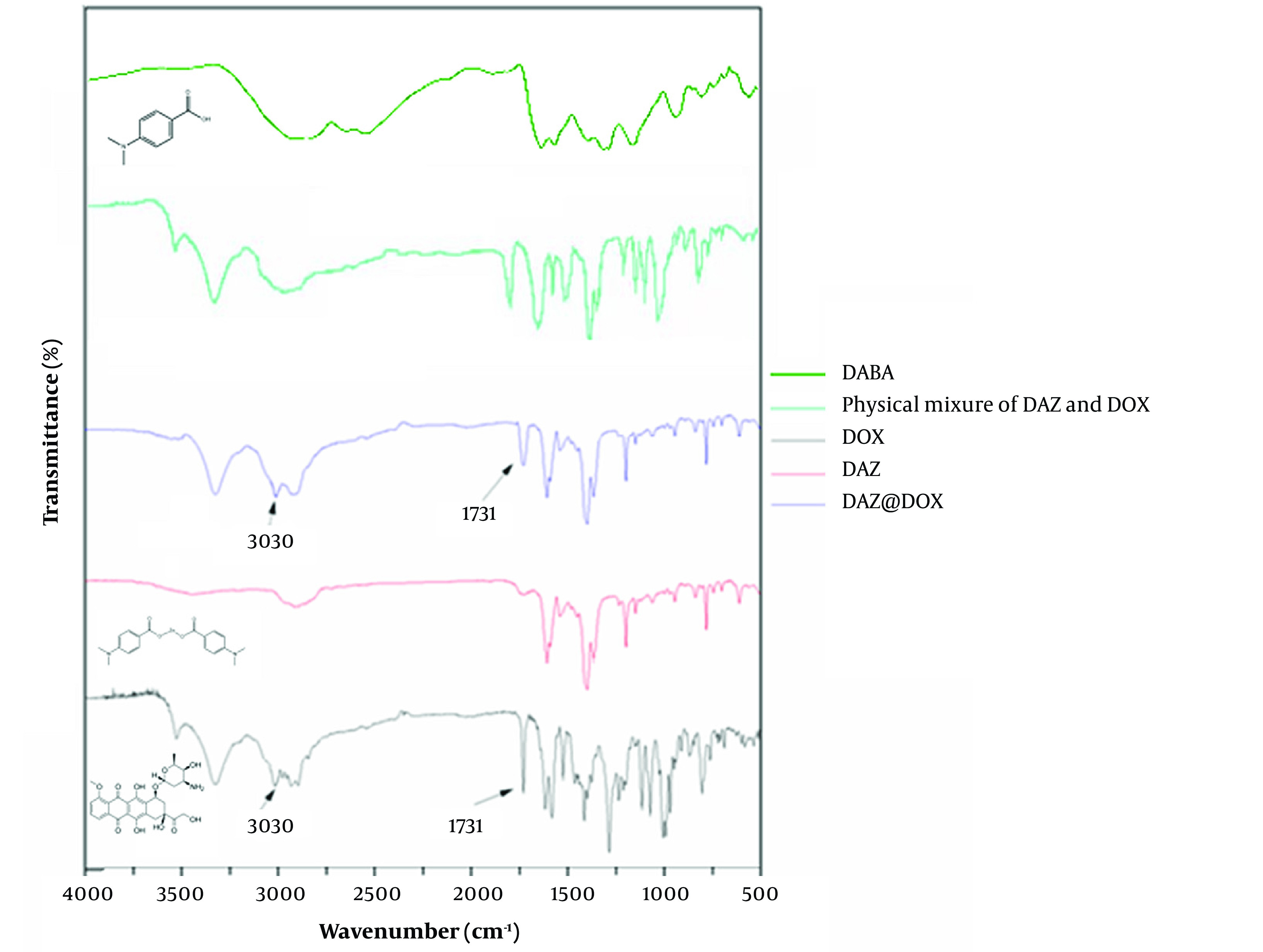
FITR spectra of DABA, physical mixture of DAZ and DOX, DOX, DAZ, and DAZ@DOX

The results of infrared spectrophotometry are presented in Figure 8. DAZ@DOX exhibited additional absorption peaks at 3030 cm^-1^ and 1731 cm^-1^ compared to DAZ alone after DOX loading. These peaks were not observed in the curve of the physical mixture of DAZ and DOX. Furthermore, the peak observed at 3030 cm^-1^ is attributed to the stretching vibration of N-H in the amino group, which is a characteristic of DOX. Similarly, the peak at 1731 cm^-1^ is generated by the skeleton stretching vibration of C=O of the carboxyl group, which also belongs to DOX. These newly emerging peaks indicate the stretching vibrations of the hydroxyl and carbonyl groups, respectively. Besides, a slight red shift occurred in the peak of the hydroxyl group from DOX, indicating that DOX and DAZ might connect by a hydrogen bond.

#### 4.1.5. UV-Vis Analysis

UV-visible absorption spectroscopy was conducted to verify the assembly of DAZ@DOX, and the result was found to be 33.74%. The results reveal that the maximum absorption peaks for DAZ and DOX were 342 nm and 463 nm, respectively. However, DAZ@DOX exhibited two maximum absorption peaks at 342 and 471 nm. These experiments demonstrated successful loading of DOX into DAZ, as evidenced by the absorption peak curves of DAZ@DOX being identical to those of DAZ and DOX. Furthermore, DAZ@DOX exhibited a slight red shift, confirming that the connections were formed within the DAZ frameworks.

**Figure 9.**
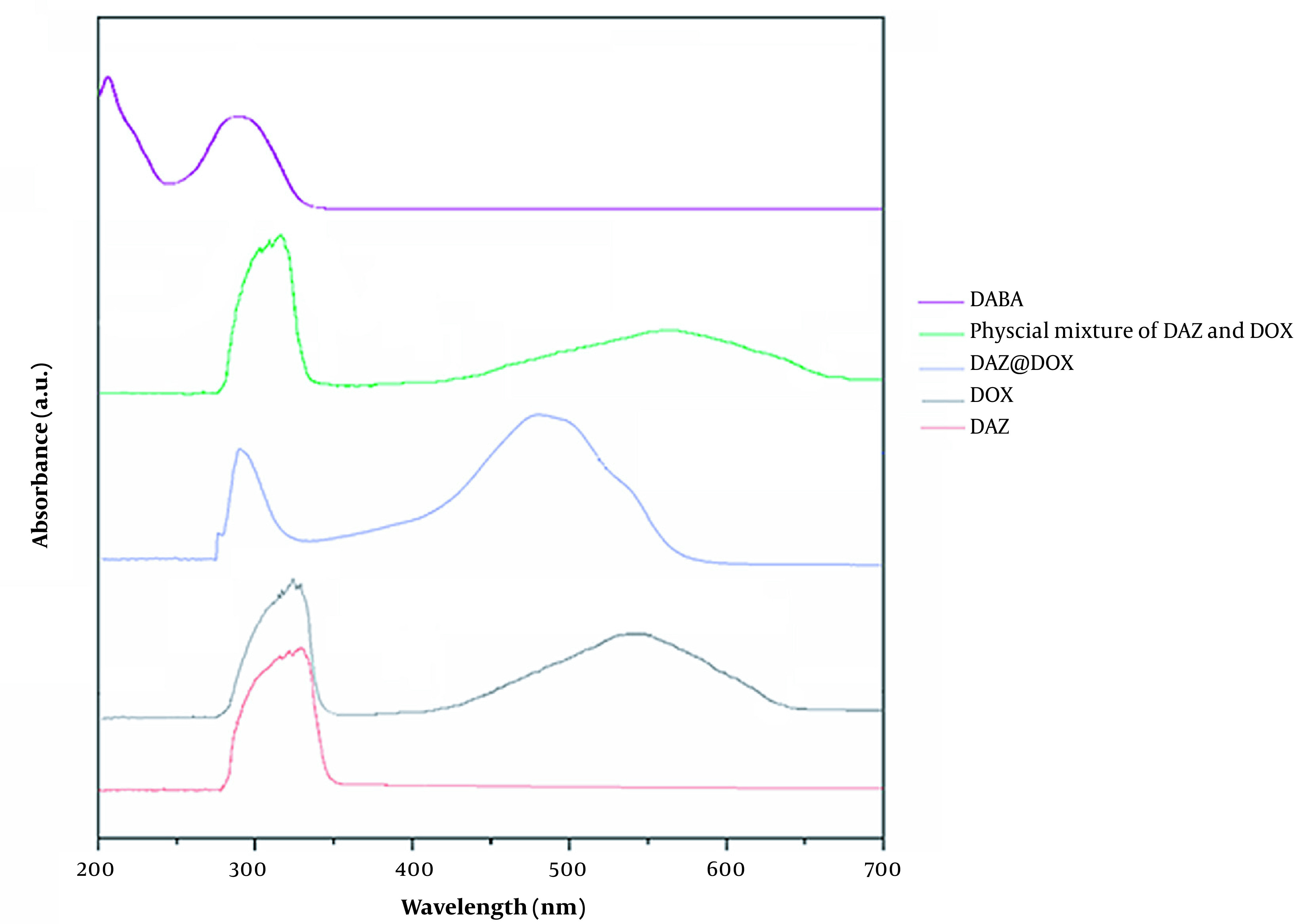
Ultraviolet-visible spectra of DABA, physical mixture of DAZ and DOX, DAZ@DOX, DOX, and DAZ

DAZ@DOX was studied using UV-vis analysis at various time intervals and ratios to investigate the impact of molar ratio and time on drug loading. As shown in Figure 9, the molar ratios of DAZ@DOX remained unchanged after 48 hours, indicating that the drug loading had reached saturation. Meanwhile, the loading amounts were highest at 24, 48, or 72 hours when the molar ratio of DAZ to DOX was 1:1. This suggests that DAZ has an optimal storage capacity.

### 4.2. Drug Delivery Properties

**Figure 10.**
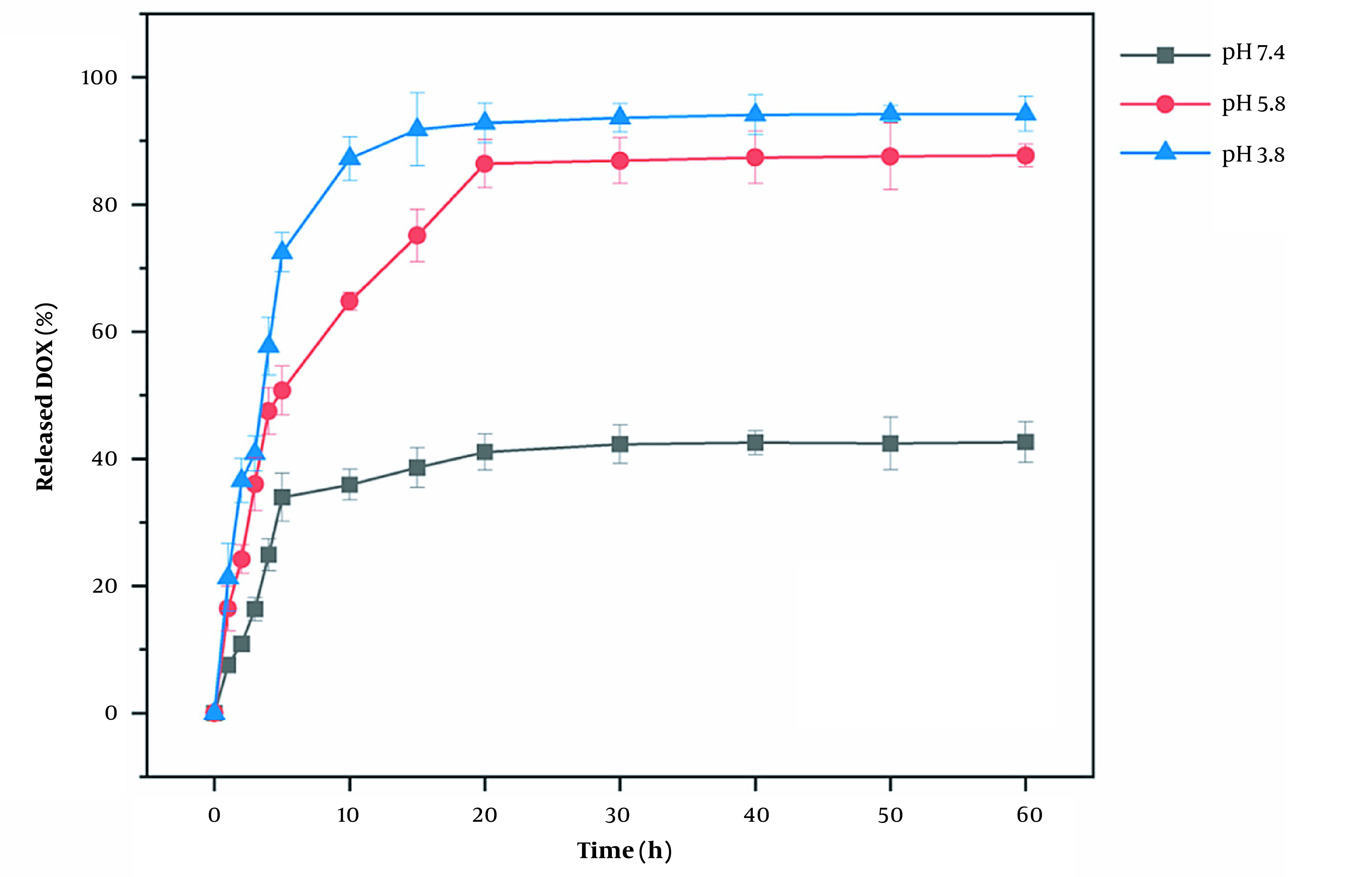
The release process of DOX from the drug-loaded DAZ

The results of the DOX drug release are shown in Figure 10. The amount of DOX released no longer changed after 15 minutes. At 15-minute intervals, the amounts of DOX released at pH 3.8, 5.8, and 7.4 were 92.17%, 55.32%, and 36.52%, respectively. These results suggest that DAZ@DOX is more effectively released at pH 3.8. In summary, DAZ@DOX exhibited pH-sensitive properties, releasing more than 90% of the drug load. This may be because the carboxylic acid groups of DAZ have a preference for accepting protons in response to changes in pH, particularly in acidic environments.

Moreover, the drug release amount at pH 3.8 was 59.62%, indicating a rapid release rate in the first five minutes, followed by a decrease in the rate after five minutes. The drug release characteristics also occur at pH 5.8 and 7.4, indicating that DAZ@DOX functions as a burst-release model for drug delivery. The entire process of DAZ@DOX was stable and fast, fulfilling the need for a drug-release carrier against cancer.

### 4.3. Cytotoxicity Test

**Figure 11.**
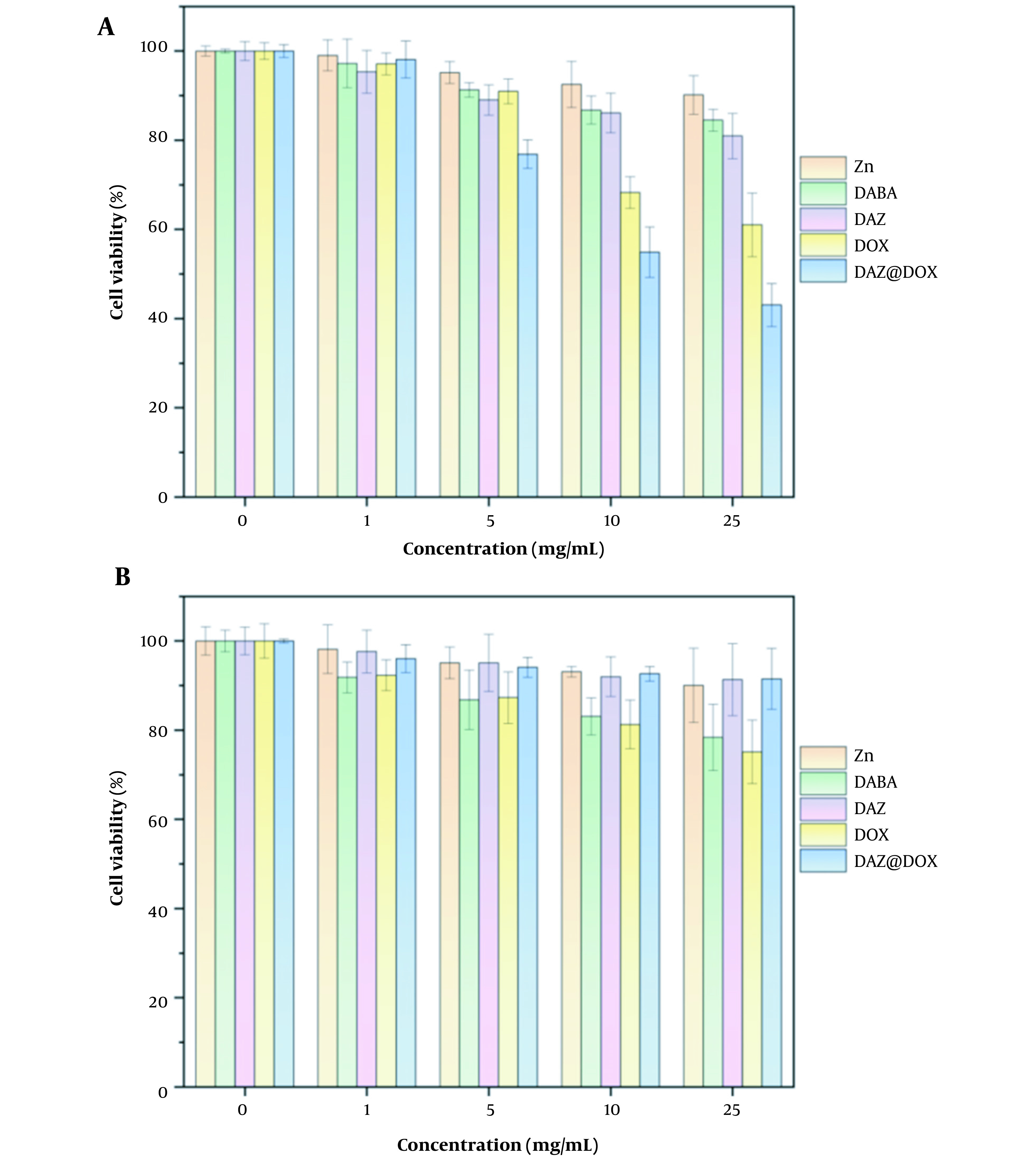
Cells viability of HepG2 and LO2 cells treated with Zn(NO_3_)_2_, DABA, DAZ, DOX, and DAZ@DOX at various concentrations for 24 h (A) HepG2; (B) LO2

The cytotoxicity of the ligand DABA, metal ions Zn^2+^, MOFs DAZ, drug DOX, and drug-loaded materials DAZ@DOX was investigated using the CCK8 method. As shown in Figure 11, DAZ@DOX exhibited no damage to normal liver cells (LO2) but demonstrated a significant anticancer effect on liver cancer cells (HepG2). Furthermore, Zn(NO_3_) and DABA did not exhibit any cytotoxic effects on HepG2 and LO2 cells. Similarly, the synthesized DAZ did not show cytotoxicity against these two cell lines. The results showed that DAZ@DOX exhibits low toxicity and high targeting toward cancer cells.

### 4.4. Conclusions

We tested the characteristics of DAZ (CDCC number: 2041878) as a drug-delivery carrier for loading DOX. A series of results revealed that DAZ has relatively good drug-loading ability, pH-sensitive characteristics, low toxicity, and selectivity. Based on these features, it is hoped that the synthesized DAZ can be used as a carrier to deliver anticancer drugs in chemotherapy for cancer to some extent. This displays potential clinical applications in the near future. Zinc-based MOFs have broad application prospects due to their excellent biocompatibility and controllable pore size. Zinc-based MOFs have the potential to achieve targeted therapy through surface modification, which can enhance drug efficacy and minimize side effects. Zinc-based MOFs have great potential as drug carriers in cancer treatment and are expected to become a crucial component of the next generation of cancer drugs.
